# Determination of basal phosphodiesterase activity in mouse rod photoreceptors with cGMP clamp

**DOI:** 10.1038/s41598-018-37661-w

**Published:** 2019-02-04

**Authors:** Teemu T. Turunen, Ari Koskelainen

**Affiliations:** Department of Neuroscience and Biomedical Engineering, Aalto University School of Science, P.O. Box 12200, FI-00076 Aalto, Finland

## Abstract

Light regulates cGMP concentration in the photoreceptor cytoplasm by activating phosphodiesterase (PDE) molecules through a G-protein signalling cascade. Spontaneous PDE activity is present in rod outer segments even in darkness. This basal PDE activity (β_dark_) has not been determined in wild type mammalian photoreceptor cells although it plays a key role in setting the sensitivity and recovery kinetics of rod responses. We present a novel method for determination of β_dark_ using local electroretinography (LERG) from isolated mouse retinas. The method is based on the ability of PDE inhibitors to decrease β_dark_, which can be counterbalanced by increasing PDE activity with light. This procedure clamps cytoplasmic cGMP to its dark value. β_dark_ can be calculated based on the amount of light needed for the “cGMP clamp” and information extracted from the registered rod photoresponses. Here we apply this method to determine β_dark_ values for the first time in the mammalian rods and obtain the following estimates for different mouse models: 3.9 s^−1^ for wild type, 4.5 s^−1^ for guanylate cyclase activating proteins (GCAPs) knockout, and 4.4 s^−1^ for GCAPs and recoverin double knockout mice. Our results suggest that depletion of GCAPs or recoverin do not affect β_dark_.

## Introduction

Photoreceptor cells convert light information to sensory signals in a process called phototransduction. When a photon is absorbed in a rhodopsin molecule in the rod outer segment disk membrane, the rhodopsin activates G-proteins, transducins, and the activated transducins bind to phosphodiesterase-6 molecules (PDE) forming enzyme complexes, which hydrolyse cyclic guanosine monophosphate (cGMP) at nearly a diffusion limited rate^1^. A rapid drop in the cytoplasmic cGMP concentration leads to the closure of the cyclic nucleotide gated (CNG) channels in the outer segment plasma membrane, hyperpolarization of the cell membrane, change in the release rate of glutamate in the rod terminal and transmission of the light-generated signal to the inner retina (see e.g.^2,3^). Thermal energy causes spontaneous activations of phototransduction molecules, which leads to fluctuations in the cytoplasmic level of cGMP. These fluctuations make up the main part the dark noise of photoreceptors^4^.

The dark noise consists mainly of three components: discrete spontaneous activations of rhodopsin, high frequency noise from fluctuations in the CNG channel conductance, and continuous noise from thermal activations of PDE^4^. The amount of active PDE in darkness determines the rate constant for spontaneous cGMP hydrolysis, i.e. the basal PDE activity (β_dark_), which sets the steady state level and the turnover rate of cGMP. Hence, it is one of the main factors in setting the kinetics of photoresponse deactivation and spatial propagation of cGMP concentration drop during photoresponses^5^. The basal PDE activity has been determined earlier for amphibian rod photoreceptors by abruptly blocking the activity of either PDE or guanylate cyclase^6–10^. In the method, single photoreceptor outer segment is exposed to rapid solution changes while recording photoreceptor circulating dark current. However, this has turned out to be challenging with the fragile mammalian photoreceptors, and until now, no one has determined the β_dark_ of wild type mammalian photoreceptors. Gross *et al*. (2012) demonstrated that when the calcium mediated feedback to guanylate cyclase is abolished by knocking out the guanylate cyclase activating proteins (GCAPs) and the lifetime of activated PDE is decreased by overexpressing RGS9, the basal PDE activity becomes the dominant factor determining the light response deactivation kinetics^5^. In these circumstances, the late recovery of a single-photon response allows the determination of β_dark_. However, it is not known whether these genetic manipulations, which affect cGMP homeostasis and PDE deactivation kinetics, influence the basal PDE activity and, thus, the β_dark_ value is not directly generalizable to wild type (WT) mice.

In this study, we introduce and test a novel approach to determine rod β_dark_ for WT mice. The method is based on the ability of PDE inhibitors to decrease both the basal and light-induced PDE activity. A decrease in the basal PDE activity can be compensated for by increasing the light-induced PDE activity in a controllable way^11–14^. As a result, the cGMP concentration in the outer segment, CNG channel current, and the photoreceptor signalling stay at constant level. We assume that the amount of light-induced PDE activity needed to keep the photoreceptor signal constant matches with the decrease in β_dark_. An essential feature in this compensation is that besides the cytoplasmic cGMP also intracellular Ca^2+^ levels stay clamped in their dark concentrations to avoid modulation of phototransduction.

In addition to WT mice, the performance of the method was investigated with GCAPs^−/−^ and GCAPs^−/−^ recoverin^−/−^ double knock out mice (DKO). The latter phenotype enables simplified modelling of photoresponses, because the predominant calcium feedbacks to phototransduction are absent^15^. The β_dark_ value determined with our cGMP clamp method was in line with the β_dark_ value obtained by modelling of GCAPs^−/−^ and DKO photoresponses, and with the value determined for GCAPs^−/−^ in^5^. The utilization of the new method is not limited to mice but it is applicable in quantitative determination of steady state phosphodiesterase activity regardless of the species or the genetic background of the model animal.

## Results

### cGMP clamp

The turnover rate and the concentration of intracellular cGMP in rod photoreceptor cells are regulated by the rates of hydrolysis of cGMP by PDE and synthesis of cGMP by guanylate cyclase. In the cGMP clamp procedure, a PDE inhibitor is introduced to the retina while monitoring changes in the extracellular voltage with local ERG recording across the outer segment layer (LERG-OS) or across the whole photoreceptors (LERG-PR). In principle, similar recordings could be conducted with transretinal ERG (TERG). However, our preliminary experiments with simultaneous TERG and LERG recordings revealed that IBMX generated an additional sustained component originating in the inner retina in spite of pharmacological blocking of synaptic transmission from rods to bipolar cells. This light-independent change in the TERG signal baseline prevented us from using TERG in cGMP clamp. A possible source for this component is the ON-bipolar cells, because IBMX has been demonstrated to potentiate the ON-bipolar cells in salamanders^16^ and in mice^17^ in light-independent manner.

**Figure 1:**
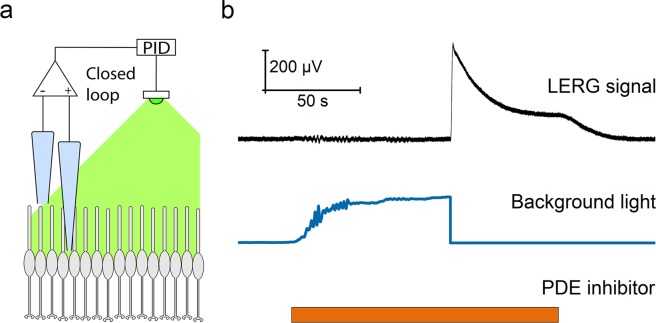
cGMP clamp method. (**a**) Illustration of background light control with recorded LERG-OS signal. The LERG-OS signal is fed to the closed loop proportional–integral–derivative (PID) controller, which adjusts the background light strength keeping the recorded signal at constant level. For clarity, the figure shows only the photoreceptor layer. (**b**) cGMP clamp recording from DKO mouse retina with LERG-OS. Background light feedback keeps the signal level constant after the introduction of PDE inhibitor (IBMX) to retina. After signal has settled to a steady state, the switch-off of the light causes a rapid increase in the recorded LERG-OS voltage.

The recorded LERG signal is proportional to changes in the circulating dark current and thus to changes in the intracellular cGMP concentration. The introduction of the PDE inhibitor decreases the catalytic activity of spontaneously (thermally) activated PDE and leads to an increase in the level of cGMP. However, the decrease in the basal PDE activity can be compensated for by increasing the amount of light-activated PDE with an accurately controlled closed loop background light feedback, thereby keeping the cGMP level clamped in the dark-adapted value (illustrated in Fig. 1). The basal PDE activity (β_dark_) can be calculated based on the light intensity needed to keep the signal stationary when the phototransduction parameters in equation (9) are known (see derivation in Methods-section).

Figure 1b presents one successful cGMP clamp run. Soon after the introduction of 40 µM IBMX, the feedback control starts to raise the background light level and the system reaches a steady state where the decline in β_dark_ is compensated with the elevated β_light_. Turning off the background light causes a rapid increase in the recorded LERG-OS voltage, reflecting the change in the cGMP level in the rod outer segments and thus in the outer segment current. However, rods cannot maintain such a high outer segment current for long and the LERG-OS voltage downregulates towards a new steady state value, probably due to the excessive energy consumption needed for retaining the elevated dark current^18^. After washout of IBMX, the signal returns to the reference level.

### Estimation of parameter values

Most of the parameter values needed for β_dark_ determination are obtained by analysing and modelling the flash responses recorded in this study. These parameters include lifetime of activated PDE, *τ*_*E*_, lifetime of activate rhodopsin, *τ*_*R*_, amplification constant for phototransduction cascade, *A*, and inhibition constants for IBMX towards light-activated, *K*_*I*,*light*_, and spontaneously activated PDE, *K*_*I*,*dark*_. In the following sections, we explain how the individual parameter values were determined for three different mouse strains, the wild type (WT), the guanylate cyclase activating protein knockout mice (GCAPs^−/−^), and the GCAPs and recoverin double knockout mice (DKO).

#### Lifetime of activated PDE

**Figure 2:**
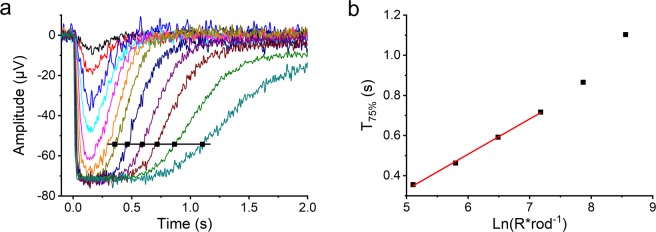
Determination of the dominant time constant τ_D_ of saturated flash response deactivation from Pepperberg plot. (**a**) LERG-OS response family from WT mouse where the time points of saturated response return to 75% level (T_75%_) are determined for the analysis. (**b**) Pepperberg plot where T_75%_ values are plotted against the natural logarithm of stimulus strength. Lifetime of active PDE is determined from the linear fit to the data. τ_D_ for this specific WT retina was 176 ms.

Pepperberg *et al*.^19^ introduced a method for determining the dominant time constant, τ_D_, of saturated flash response deactivation, which is demonstrated in Fig. 2 for one example WT retina. In their analysis, the kinetics of saturated response recovery is assumed to be controlled by a single first order deactivation reaction causing the time that responses spend in saturation to increase linearly with respect to the natural logarithm of flash stimulus strength. The slope of the increasing saturation time determines the time constant, which is considered to represent the average lifetime of light-activated PDE for mouse rods^20^. The average τ_D_ estimates obtained with the Pepperberg analysis were 191 ± 13 ms (n = 10) for WT, 222 ± 7 ms (n = 6) for GCAPs^−/−^, and 200 ± 13 ms (n = 9, mean ± SEM) for DKO mice.

#### Amplification constant and lifetime of activated rhodopsin

In Lamb and Pugh activation model^21^, the response onset is assumed to be defined only by the activation phase of the phototransduction and it can be fitted to early onset of the responses until the response termination starts to affect the response shape. In mouse rods, the shortness of the rhodopsin lifetime is the main factor limiting the valid time window for Lamb and Pugh activation model fitting because coincidentally with the deactivation of rhodopsin, the steepness of the photoresponse onset starts to decline. Deactivation of rhodopsin proceeds through a sequence of several phosphorylation steps by rhodopsin kinase, each of which increases the probability for an arrestin molecule to bind and completely deactivate the activated rhodopsin^22–25^. Determination of the average lifetime of active rhodopsin (*τ*_*R*_) has turned out to be challenging and currently there are no means for its direct determination in mammalian photoreceptors. Nevertheless, *τ*_*R*_ can be estimated by modelling rod photoresponses. With the simplifying assumption that rhodopsin deactivation follows first order reaction kinetics on average, the mean lifetime for rhodopsin in WT mouse rods is estimated to lie close to 40 ms^26^. This proposes that the rate of transducin activation will drop to half in less than 30 ms from a brief stimulus, leaving only a very narrow time window to determine the “true” amplification constant of phototransduction before rhodopsin deactivation substantially starts to shape the responses.

**Figure 3:**
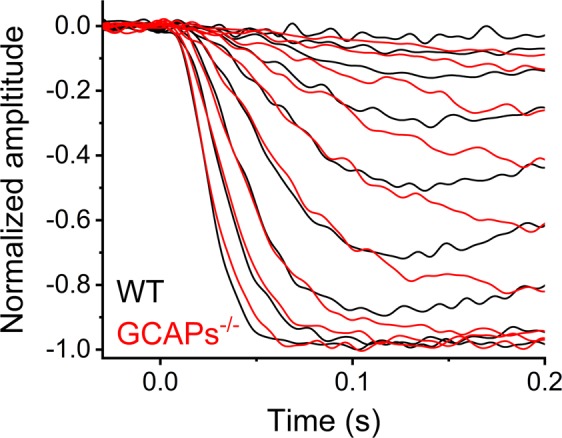
Population averaged response families from 10 WT and 6 GCAPs^−/−^ mouse retinas recorded in LERG-OS geometry. The average flash strengths for WT response family were 1.7, 3.0, 5.9, 12, 24, 48, 95, 190, and 370 R*rod^−1^. The average flash strengths for GCAPs^−/−^ response family were 1.4, 2.8, 5.5, 11, 22, 44, 87, 170, and 350 R*rod^−1^.

To extend the time window for the determination of the amplification constant, we used a model that takes into account the activation reactions as well as deactivation of activated rhodopsin and PDE but disregards the hydrolysis of cGMP by basal PDE activity and synthesis of cGMP by guanylate cyclase (equation (17)). Equation (17) is valid only (1) when changes in guanylate cyclase activity are minor, i.e. Δ*α*(*t*, *Ca*^2+^) ≈ 0, and (2) when the following holds: *β*_*light*_(*t*)*cGMP*(*t*) ≫ *β*_*dark*_(*cGMP*_*dark*_ − *cGMP*(*t*)) (see Methods for derivation). The first condition holds always with GCAPs^−/−^ and DKO mice, but for WT mice it is true only for the early activation phase of the response. This is demonstrated in Fig. 3 which shows population averaged response families from WT (n = 10) and GCAPs^−/−^ (n = 6) retinas recorded with LERG-OS. The activation phases of the responses to similar flash strengths start to diverge clearly only after 70–100 ms from the response onset as shown earlier for dim flash responses^27,28^. So, we assume in our analysis that condition 1 holds at times <70 ms after the flash.

**Figure 4:**
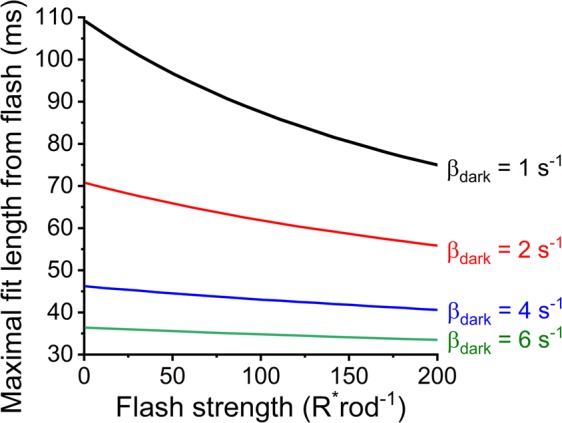
Testing the validity of the model for determining the amplification constant and rhodopsin lifetime (equation (17)). Model was assumed to be valid from the time point of the flash stimulus until the time the error made from the model assumptions would be greater than 10%. This range defines the maximal fit length from flash (y-axis). Parameters used for modeling were A = 20 s^−2^, τ_E_ = 200 ms, τ_R_ = 20 ms, n_cGMP_ = 3, α_dark_ = 16.7 µMs^−1^, and constant time delay of 7 ms. cGMP concentration in darkness was calculated as cGMP_dark_ = α_dark_/β_dark_. β_light_ was increased by varying the flash stimulus strength from 1 to 200 R*rod^−1^. This range enclose responses from single photons to rod saturating stimuli.

The fulfilment of the condition (2) depends on both the light-induced and basal PDE activity levels, *β*_*light*_(*t*) and *β*_*dark*_. We set the criterion for the validity of condition (2) to *β*_*light*_(*t*)*cGMP*(*t*) > 10⋅*β*_*dark*_(*cGMP*_*dark*_ − *cGMP*(*t*)). We then calculated the maximal time from the flash stimulus that still meets the criterion. To analyse the condition, we calculated *β*_*light*_(*t*) with different flash strengths with equations (13) and (14). The parameters needed to estimate *β*_*light*_(*t*) were chosen to be realistic but at the same time to give rather too narrow than wide range for the validity of equation (17). The parameters *ν*_*RE*_ and *β*_*sub*_ can be combined to their product, whose value can be obtained from equation (8) by determining the amplification constant *A*. The value of Hill’s coefficient for CNG channels was taken from literature, *n*_*cGMP*_ = 3^2,5^. In our analysis, the highest amplification constant value was about 20 s^−2^ and we used this value in the analysis. Higher values would narrow down the validity range of equation (17). 200 ms was used as the lifetime of activated PDE *τ*_*E*_, *i.e. τ*_*D*_, which is close to *τ*_*D*_ determined for the studied mouse strains. We used 20 ms as our lowest estimate for rhodopsin lifetime, *τ*_*R*_, a value that is expected to be below or close to the minimum value of *τ*_*R*_. *cGMP*(*t*) was calculated numerically from equation (16) and *cGMP*_*dark*_ was derived from the relation *cGMP*_*dark*_ = *α*_*dark*_/*β*_*dark*_ with *α*_*dark*_value of 16.7 µMs^−1^ ^5^. Further, we used a constant time delay, *t*_*delay*_, of 7 ms for the sum effect of all delays in the phototransduction machinery and recording equipment. The analysis was conducted for *β*_*dark*_ values ranging from 1 to 6 s^−1^ and with flash strengths from 1 to 200 R*rod^−1^ which is enough to cover the operation range of dark-adapted mouse rods. The results are shown in Fig. 4. When *β*_*dark*_ is expected to be as high as 6 s^−1^ and flash strength reaches 200 R*rod^−1^, the error made with condition (2) is less than 10% during the first 34 ms from the beginning of the flash response. The validity time set by the second criterion, 34 ms, is shorter than the one set by the first criterion, 70 ms, and thus, the fitting of the phototransduction model was carried out using only the first 34 ms of the responses from the beginning of the flash stimulus. If our final *β*_*dark*_ estimate was higher than 6 s^−1^, this analysis should be repeated with a tighter criterion for the validity of equation (17).

**Table 1:** Parameter values used for modeling of fractional dim flash responses from GCAPs^−/−^ (Fig. 8b) and DKO (Fig. 8d) mice recorded with LERG-OS geometry.The dominant time constant τ_D_ was determined separately from each experiment from Pepperberg plot and the mean value was used as τ_E_ in the modelling.

Parameter		Units	GCAPs^−/−^	DKO
Fit length	—	ms	600	600
Constant time delay	t_delay_	ms	7	7
Lifetime of R*	τ_R_	ms	49.0	28.5
Lifetime of PDE*	τ_E_	ms	203	196
Amplification constant	A	s^−2^	15.7	18.8
Basal PDE activity	β_dark_	s^−1^	4.51	4.45
Synthesis rate of cGMP	α_dark_	µM·s^−1^	16.7	16.7
cGMP level in darkness	cGMP_dark_	µM	3.70	3.75
The Hill coefficient for CNG channels	n_cGMP_	—	3	3

**Figure 5:**
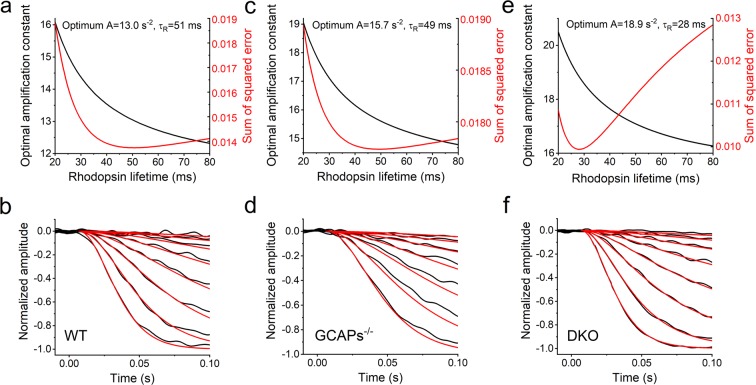
Determination of rhodopsin lifetime by fitting the model utilizing equation (17) to the activation phase of population averaged response families recorded with LERG-OS with 2-fold increments in flash strengths. (**a,c,e**) Presents the fitting outcome with different τ_R_ values to WT, GCAPs^−/−^, and DKO response families, respectively. Black curves show the optimal amplification constants with different τ_R_ values and red curves show the sum of squared errors of the fits. The optimal τ_R_ values are found from the minima of the red curves. (**b,d,f**) Show the WT, GCAPs^−/−^, and DKO response families, respectively, and the model fittings with the optimal τ_R_ and A values. (**b**) Response families from 10 WT experiments with flash strengths ranging from 1.7 to 187 R*rod^−1^. The optimal τ_R_ was 51 ms and the subsequent amplification constant was 13.0 s^−2^. (**d**) Response families from 6 GCAPs^−/−^ experiments with flash strengths ranging from 1.4 to 87 R*rod^−1^. The optimal τ_R_ was 49 ms and the amplification constant was 15.7 s^−2^. (**f**) Response families from 18 DKO experiments with flash strengths ranging from 1.4 to 170 R*rod^−1^. τ_R_ of 28 ms and amplification constant of 18.9 s^−2^ gave the optimal fit to the responses. With all mouse strains, the fitting was performed for responses ranging from dim flashes to the first saturated response.

To maximize the signal-to-noise ratio in our fitting of the phototransduction model to the early phase of light responses, we calculated population averaged flash response families for WT, GCAPs^−/−^, and DKO mice, respectively, from all registrations recorded with LERG-OS. Since in the fitting, amplification constant *A* and rhodopsin average lifetime *τ*_*R*_ cannot be determined fully independently from each other, we fitted the model with all possible combinations of *A* and *τ*_*R*_. The lifetime of PDE was locked to the values determined as described earlier. Figure 5 shows the least square fits to the WT, GCAPs^−/−^ and DKO population averaged responses recorded with LERG-OS. Figure 5a,c,e illustrate the sum of squared error of the fitting with different rhodopsin lifetimes and the conjoined optimal amplification constant values. The optimal fits were achieved with rhodopsin lifetimes of 51 ms for WT, 49 ms for GCAPs^−/−^, and 28 ms for DKO mice. From now on, the rhodopsin lifetimes were fixed to these estimated values and the amplification constants were determined separately for each retina used in the cGMP clamp experiments. The average amplification constants were 15.8 ± 1.8 s^−2^ for WT (n = 10), 14.0 ± 1.6 s^−2^ for GCAPs^−/−^ (n = 6), and 20.6 ± 2.6 s^−2^ for DKO mice (n = 9, mean ± SEM). These values are close to the amplification constant values determined earlier for mouse rods with suction electrode recordings in resembling nutrition medium (range from 8.3 to 23 s^−2^)^29–31^.

#### Inhibition constants for IBMX

**Figure 6:**
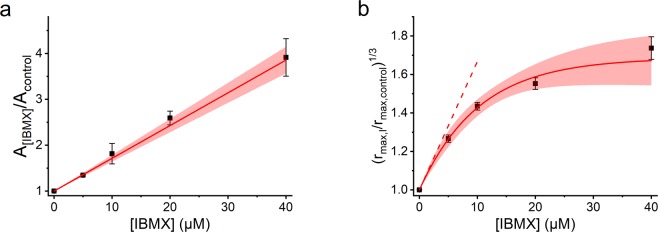
Determination of inhibition constant towards light activated and basally active PDE for IBMX. (**a**) Determination of inhibition constant towards light activated PDE (K_I,light_). Fit to the pooled data for DKO mice (n = 9) gave an inhibition constant of 14.0 ± 0.5 µM (mean ± SER). The intercept of the linear fit is forced to 1. (**b**) Determination of inhibition constant towards basally activated PDE (K_I,dark_). Increase in dark voltage gets downregulated with high IBMX concentrations. The figure shows the cubic root of the relative increase in dark voltage right after the light turn off in cGMP clamp (black squares) for pooled data from DKO mice (n = 9). The exponential fit to the cGMP clamp data gives extrapolated value of 15.0 ± 1.1 µM (mean ± SER) for K_I,dark_. The filled areas under the fits presents 95% confidence limits.

The inhibition constant of IBMX towards light-activated PDE (*K*_*I*,*light*_) was determined based on the decrease in phototransduction amplification caused by the PDE inhibitor as described in^32^. The inhibition constant was determined separately for each retina and the average *K*_*I*,*light*_ for IBMX was 16.3 ± 1.0 µM for WT (n = 10), 13.2 ± 1.2 µM for GCAPs^−/−^ (n = 6), and 13.8 ± 1.7 µM for DKO (n = 9, mean ± SEM) mice. Figure 6a demonstrates the *K*_*I*,*light*_ determination from averaged DKO data.

For IBMX, the inhibition constant towards light-activated and spontaneously activated PDE have been found to be very similar in literature^13,32^. We verified this by determining the *K*_*I*,*dark*_ value from the increase in maximal LERG-OS amplitude after the light turn off in cGMP clamp (see Fig. 1) with the method described in^32^. The inhibition constant was then obtained from the equation (equation (15) in^32^)

1 $${(\frac{{r}_{max,I}}{{r}_{max,control}})}^{1/{n}_{cGMP}}=\frac{[I]}{{K}_{I,dark}}+1.$$

The *K*_*I*,*dark*_ determination was done by extrapolating the increase in the relative maximal LERG-OS signal to zero inhibitor concentration. Because the increase in the outer segment current (monitored by the LERG-OS signal) is modulated by intracellular calcium, the *K*_*I*,*dark*_ determination was accomplished with the DKO mice with the calcium-dependent feedback mechanisms knocked out. When the data from individual experiments (n = 9) were pooled together, the *K*_*I*,*dark*_ obtained for the DKO mice was 15.0 ± 1.1 µM (mean ± standard error of regression, SER) (Fig. 6b). The *K*_*I*,*dark*_ values did not differ significantly from the *K*_*I*,*light*_ values, letting us to use the *K*_*I*,*light*_ value determined separately for each retina as the common inhibition constant for both light-activated and spontaneously activated PDE.

### Determination of basal PDE activity by cGMP clamp

**Figure 7:**
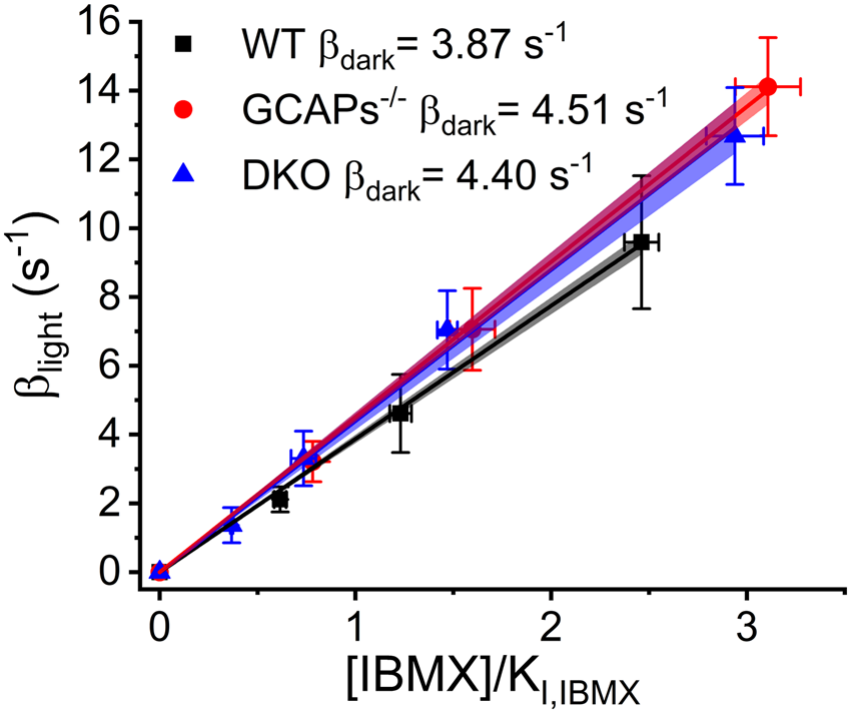
Determination of β_dark_ from cGMP clamp data. The linear fits to the data according to equation (10) gave β_dark_ – values of 3.87 ± 0.04 s^−1^ (n = 10, mean ± SER) for WT, 4.51 ± 0.09 s^−1^ (n = 6, mean ± SER) for GCAPs^−/−^ and 4.40 s^−1^ ± 0.04 (n = 9, mean ± SER) for DKO mouse. The linear fit was forced to pass through the origin. The error bars presents SEMs and the filled areas under the linear fits presents 95% confidence limits.

After specifying the parameter values needed in equation (9), we determined β_dark_ from the collected cGMP clamp data. Figure 7 shows the population average of light-activated PDE activity plotted against IBMX concentration normalized by its inhibition constant. The β_dark_ values were obtained from the slopes of the fitting of a linear fits to the plotted cGMP clamp data. The β_dark_ values were 3.87 ± 0.04 s^−1^ for WT, 4.51 ± 0.09 s^−1^ for GCAPs^−/−^, and 4.40 ± 0.04 s^−1^ for DKO mice (mean ± SER). With DKO mice, local ERG was recorded across the rod outer segment layer, while with WT and GCAPs^−/−^ mice the LERG recordings were conducted across the whole photoreceptor layer to improve the signal-to-noise ratio. The reason for using LERG-OS with the DKO mice was that the flash response data from the same experiments was used for modelling of flash responses (see below). Our results indicate that there is no significant difference in the β_dark_ values between the tested mouse strains.

### Sensitivity of β_dark_ for chosen parameter values

**Figure 8:**
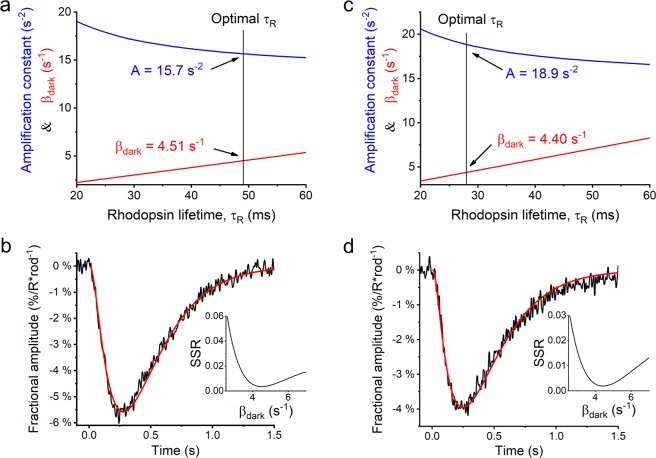
Testing the cGMP clamp method sensitivity by modeling GCAPs^−/−^ (n = 6) and DKO (n = 18) fractional dim flash responses recorded by LERG-OS. (**a,c**) Dependencies of parameters τ_R_, A, and β_dark_ from each others with GCAPs^−/−^ and DKO mice, respectively. The blue and red traces show the values for A and β_dark_ as τ_R_ is changed gradually from 20 ms to 60 ms. The vertical lines highlight the optimal τ_R_ and A values from activation phase fits (see Fig. 5) and the subsequent β_dark_ values from cGMP clamp. (**b,d**) Optimal fit to the whole fractional dim flash responses from GCAPs^−/−^ and DKO mice, respectively. The modelling parameters are displayed in Table 1. The inset shows how the sum of squared error (SSR) of the fit behaves when the fitting parameters are tuned from the optimum.

When modelling the early activation phase of the flash responses, small changes in the rhodopsin lifetime can be compensated for with opposing changes in the amplification constant without causing major increase in the sum of squared error of the fit (see Fig. 5). However, choosing another τ_R_ value in the vicinity of the optimum will change the β_dark_ value determined from cGMP clamp, because β_dark_ is proportional to A and τ_R_ (see equation (9)). The dependence of β_dark_ on different combinations of A and τ_R_ are shown in Fig. 8a,c for GCAPs^−/−^ and DKO, respectively.

To estimate which of these parameter combinations are plausible, we tested how well prospective parameter triplets (τ_R,i_, A_i_, β_dark,i_) can describe the rod flash response behaviour. We modelled the fractional dim flash responses of GCAPs^−/−^ and DKO mice, where the fractional responses were obtained by dividing the dim flash response with the saturation amplitude and the flash strength to give estimates for the sizes of fractional single photon responses. With GCAPs^−/−^ and DKO mouse strains, the dim flash responses are not significantly modulated by calcium^15,27^. Therefore, we can apply equation (16) to model the whole fractional responses as described in Methods. Figure 8b,d display the model curves with the parameter triplets that gave the best fits to the fractional dim flash responses of GCAPs^−/−^ and DKO mice, respectively. For GCAPs^−/−^ the best parameter values were τ_R_ = 49.0 ms, A = 15.7 s^−2^, β_dark_ = 4.5 s^−1^, and for DKO τ_R_ = 28.5 ms, A = 18.8 s^−2^, β_dark_ = 4.5 s^−1^. Table 1 presents the optimal fitting parameters as well as the parameter values taken from the literature and kept constant in the modelling (*α*_*dark*_ and *n*_*cGMP*_). The insets in Fig. 8b,d illustrate how the sum of squared error of the fit (SSR) increases when the parameter triplets recede from the optimum. For clarity, the x-axis only shows the β_dark_ value and not the conjoined τ_R_ and A. With both mouse strains, the optimal β_dark_ value coincided with that determined by cGMP clamp.

## Discussion

The basal PDE activity (β_dark_), together with guanylate cyclase activity (*α*), determines the turnover rate and the intracellular concentration of cGMP in photoreceptors in darkness. Rieke and Baylor concluded that the natural variation in basal PDE activity in toad photoreceptors is the main source of the fluctuations in the intracellular cGMP concentration in darkness^4^ and that β_dark_ is a key factor in setting the rod sensitivity and the temporal resolution of rod signalling in the dark adapted state. Still, no one has determined the rate constant for spontaneous cGMP hydrolysis in the small mammalian wild type photoreceptors. A pioneering effort to this direction was taken by Gross, Pugh, and Burns (2012), whose analysis was based on the assumption that the time constant determining the very latest recovery phase of dim flash responses is set by β_dark_ in conditions, where the other time constants of phototransduction deactivation (*τ*_*R*_ and *τ*_*E*_) are short compared to 1/β_dark_, and where there is no calcium feedback to response recovery^5^. These circumstances can be obtained in GCAPs^−/−^ mice when *τ*_*E*_ is significantly shortened by overexpressing the RGS9 complex. Based on the time constant of the final recovery phase of dim flash responses, they estimated β_dark_ to be 4.1 s^−1^ in GCAPs^−/−^ background. However, the method is not valid for WT mice, in which the calcium-mediated feedback to guanylate cyclase activity accelerates the response recovery. For comparison, the basal PDE activity estimates vary from 0.1 s^−1^ to 1.5 s^−1^ in toad rods^4,33,34^, from 0.49 to 3.4 s^−1^ in frog rods^7,35^, and from 1.2 to 2.8 s^−1^ in salamander rods^6,8,13^ at room temperature.

In this study, we introduced a method for β_dark_ determination that allowed us to determine the basal PDE activity for WT mice for the first time. The use of cGMP clamp requires knowledge of the values of several phototransduction parameters, some of which are hard to determine. E.g. there is no direct electrophysiological method for determination of the Hill’s coefficient for the CNG channel activation (*n*_*cGMP*_) or the time constant for deactivation of rhodopsin activity (τ_R_). The *n*_*cGMP*_ value is generally accepted to lie close to 3 for mouse rod photoreceptors^2,5^ and this value was used in the analysis. Larger uncertainty arises from the determination of τ_R_. When analysing the lifetime of activated rhodopsin and the amplification constant A by modelling the early onset phase of flash responses, A and τ_R_ appear mutually dependent: a change in either of the parameter values requires an opposite shift in the other to achieve a reasonable fit (see Fig. 5). Because A and τ_R_ appear as a product in equation (9), the shift in β_dark_ value determined with cGMP clamp is gentler than the relative change in the values of either A or τ_R_. Still, if τ_R_ were forced to 20 ms for the DKO mice, the amplification constant would be increased only to 20.6 s^−2^ for a suitable fit. With these parameter values, the β_dark_ obtained from cGMP clamp would be 3.4 s^−1^ which is somewhat smaller than that achieved with the best fit (τ_R_ = 28 ms, A = 18.9 s^−2^, and β_dark_ = 4.4 s^−1^). To obtain further support for our β_dark_ determination by cGMP clamp, we modelled dim flash responses recorded from GCAPs^−/−^ and DKO mouse retinas while restricting the parameter combinations to those that are plausible according to the activation phase modelling and cGMP clamp results (see Fig. 8). The best fits were obtained with almost identical values compared to those determined by cGMP clamp.

In these analyses, rhodopsin activity was assumed to decay with a single exponential time constant, τ_R._ However, the mechanistic view is that rhodopsin deactivation proceeds through several phosphorylation steps, which decrease the activity of rhodopsin and increase the probability of arrestin binding in a stepwise manner^22,23,25,33,36–41^. The implication of replacing the multistep rhodopsin deactivation with exponential decay of rhodopsin activity in phototransduction modelling was considered in^41^. The very beginning of the activation phase of the responses behaves closely similarly in both models but soon the exponential model starts to slightly overestimate the rhodopsin activity (see Fig. S2 from^41^). In our simultaneous determination of activation constant and rhodopsin lifetime (see Fig. 5), this overestimation is at least partly compensated in the amplification constant determination. *A* has to be set slightly too low in order to fit the model to beginning of flash responses when rhodopsin activity is estimated higher than appropriate. Additionally in dim flash response modelling in GCAPs^−/−^ background (Fig. 8), the response deactivation kinetics are set mainly by τ_E_ and β_dark_ but not affected by τ_R_. Thus, we believe that the effect from assuming exponential decay of rhodopsin activity is small in our β_dark_ determination. Moreover, late studies have indicated that rhodopsin activity might not decrease in graded fashion but instead rhodopsin goes through a low activity state before arrestin binding^24^. This leads to a small delay before decrement of rhodopsin activity which is not regarded either in the exponential model or in the multistep deactivation model of rhodopsin^41^. The mechanism and its implication for rhodopsin deactivation still needs further consideration.

As illustrated in Fig. 7, our β_dark_ values for WT (3.9 s^−1^) and for GCAPs^−/−^ (4.5 s^−1^) mice do not seem to differ significantly. This is somewhat presumable because the expression level of PDE or the dark current level is not expected to change due to these genetic modifications^42,43^. Further, these values are in good agreement with that determined earlier for GCAPs^−/−^ mice with a completely different approach (4.1 s^−1^)^5^. Therefore, it seems very reliable to conclude that the β_dark_ value for WT mouse rods (as well as for GCAPs^−/−^ mice rods) is close to 4 s^−1^. On the other hand, it has been proposed that the basal phosphodiesterase activity in mouse rods might be directly modulated by recoverin^44^. Our results suggest that removal of GCAPs (in the presence of recoverin) or recoverin (in the absence of GCAPs) do not significantly affect β_dark_. However, the essential feature of our cGMP clamp method is that the cGMP and Ca^2+^ levels are maintained at their dark adapted levels and therefore our results do not rule out the possibility that recoverin might modulate β_dark_ as a result of changes in the intracellular calcium concentration. Furthermore, the novel method will allow determination of β_dark_ value in cones, which likely differs from that in rods^45^, as well as investigation of potential modulation of β_dark_ by factors such as glutamic acid-rich protein-2^46^ or intracellular calcium concentration^15^. Overall, the introduced method offers a means to examine β_dark_ in different animal models by recording ERG signals from the isolated retina, a situation closely resembling to *in vivo* condition.

## Methods

### Experimental

#### Ethical approval

The use and handling of the animals were in accordance with the Finnish Act on Animal Experimentation 2006 and guidelines of the Animal Experiment Board in Finland. Experimental protocols were approved by Laboratory Animal Centre of the University of Helsinki, and by the Animal Experimental Board in Finland. In addition, Board for Gene Technology in Finland has approved our laboratory for the use of gene-manipulated mice.

#### Animals and preparation

Wild type (C57BL/6J), GCAPs^−/−^, and GCAPs^−/−^ recoverin^−/−^ double knockout (DKO) mice, derived from GCAPs^−/−^ and recoverin^−/−^ mice^42^ kindly provided by Dr. Jeannie Chen (University of Southern California), were used in this study. Mice were dark adapted overnight and sacrificed by CO_2_ inhalation followed by cervical dislocation. The eyes were enucleated and small incisions were made along the equators of the eyes. The eyes were bisected by enlarging the incision with micro scissors and the isolated eyecup was placed into cooled nutrition medium (composition described in Recording chamber, the recordings and perfusion-section). One eyecup was stored at +7 °C in nutrition medium in a light tight container to be used later during the same day. The retina was removed from the eyecup with forceps and micro scissors under a microscope and the whole retina was placed in a specimen holder into a light-tight Faraday cage. The procedures described above were completed under a dim red light.

#### Recording chamber, the recordings and perfusion

Our specimen holder allows simultaneous visualization, stimulation and perfusion of the retina, and it is equipped with an open passage for microelectrodes enabling local electroretinography recording^47^. Local electroretinography (LERG) were recorded across the rod outer segment layer (LERG-OS) or across the whole photoreceptors (LERG-PR). The LERG-OS signal is directly proportional to the changes in the rod outer segment current^47^. The recording electrode (with a tip diameter ∅ of 2–5 µm) was passed to the depth of ~25 µm (OS) or ~100 µm (PR) in the retina and the reference electrode (tip ∅ ~ 30 µm) was located on the surface of the retina. The surface was identified both visually and from the voltage shift observed when the recording electrode penetrated the surface of the retina. During the LERG recordings, simultaneous transretinal ERG recordings were conducted with macroelectrodes located on both sides of the retina. The recording geometry is described more in detail in^47^.

The open chamber of the specimen holder was filled with nutrition medium and a laminar flow of medium perfused the photoreceptor side of the retina with a constant rate (*ca*. 3 ml/min). The composition of the nutrition medium was (mM): Na^+^, 133.4; K^+^, 3.3; Mg^2+^, 2.0; Ca^2+^, 1.0; Cl^−^, 143.2; glucose, 10.0; EDTA, 0.01; HEPES, 12.0, adjusted to pH 7.5 with 5.8 mM NaOH. The viability of the retina was improved by adding 0.72 mg/ml Leibovitz culture medium L-15 to the solution. Synaptic transmission from photoreceptor cells to bipolar cells was blocked by adding 2 mM sodium aspartate^48^. To abolish the glial component arising from Müller cells, 50 µM BaCl_2_ was added to the nutrition medium^48,49^. 3-isobutyl-1-methylxanthine (IBMX) was used as a PDE inhibitor at concentrations of 5, 10, 20 and 40 µM. All chemicals were purchased from Sigma-Aldrich (Espoo, Finland).

The specimen holder was placed on top of a heat exchanger whose temperature could be controlled with water circulating heating bath (LTD6G; Grant Instruments Ltd, Shepreth, Royston, UK). Recordings were conducted at physiological temperatures 37 ± 1 °C. The temperature in the nutrition medium close to the retina was monitored continuously with a calibrated thermistor (30K6A309I; BetaTHERM; Measurement Specialties, Inc., Hampton, VA, USA).

#### Light stimulation

Light stimulation was accomplished with 1 ms flashes from a LED light source (Luxeon Rebel LXML-PM01-0100, λ_max_ = 532 nm; Lumileds, Amsterdam, Netherlands). The stimuli illuminated the whole retina homogeneously as verified with a camera-based beam profiler (Spiricon Laser Beam Diagnostics Model SP503U). The absolute light intensity incident on retina was measured with a calibrated photodiode (Thorlabs GmbH FDS100-cal). The number of rhodopsin isomerizations (R*rod^−1^ or R*rod^−1^ s^−1^) caused by the stimulus was calculated based on the LED and photodiode spectra, respectively, and the pigment template by Govardovskii *et al*. (2000) as described in Heikkinen *et al*.^50^.

A proportional–integral–derivative (PID) controlled closed loop feedback from the recorded ERG voltage signal to the light source was developed in order to keep the recorded signal constant when the PDE inhibitor was introduced to the retina. The light control feedback was carried out digitally with LabVIEW (National Instruments, Austin, TX, USA).

#### Data acquisition

Data acquisition and LED controls were handled with a data acquisition card (PCIe-6351; National Instruments) and a custom made LabVIEW software. The recorded DC signal was sampled at 1000 Hz with a voltage resolution of 15 nV and amplified 1000-fold. The signals were low-pass filtered at f_c_ = 500 Hz (8-pole Bessel filter) and afterwards digitally at f_c_ = 100 Hz.

### Theoretical background and calculations

#### cGMP clamp

The following section introduces the theoretical background of the cGMP clamp method with help of phototransduction equations. The analysis is based on the phototransduction models described thoroughly in^2^.

In background light, phototransduction reactions can be assumed to obey first order reaction kinetics:

2 $$\{\begin{array}{c}\frac{d{R}^{\ast }(t,\,C{a}^{2+})}{dt}={\Phi }_{BG}(t)-{k}_{R}{R}^{\ast }(t,\,C{a}^{2+})\\ \frac{dPD{E}^{\ast }(t,\,C{a}^{2+})}{dt}={\nu }_{RE}{R}^{\ast }(t)-{k}_{E}PD{E}^{\ast }(t,\,C{a}^{2+})\\ \frac{dcGMP(t,\,C{a}^{2+})}{dt}=\alpha (t,\,C{a}^{2+})-\beta (t,\,C{a}^{2+})cGMP(t)\\ =\,\alpha (t,\,C{a}^{2+})-({\beta }_{dark}+{\beta }_{light}(t,\,C{a}^{2+}))cGMP(t)\\ =\,\alpha (t,\,C{a}^{2+})-({\beta }_{dark}+{\beta }_{sub}PD{E}^{\ast }(t,\,C{a}^{2+}))cGMP(t),\end{array}$$

where R^*^ is the number of active rhodopsin molecules, PDE^*^ the number of active PDE subunits, *Φ*_*BG*_ is the number of activated rhodopsin molecules per second in one rod by the background light [*Φ*_*BG*_] = R*rod^−1^ s^−1^, *k*_*R*_ and *k*_*E*_ are rate constants for rhodopsin and PDE deactivation. *α* is the rate of cGMP synthesis and *β* is the rate constant of cGMP hydrolysis. *β*_*dark*_ and *β*_*light*_ are the hydrolysis rate constants of cGMP by basally active and light-activated PDEs, respectively. *β*_*sub*_ is the hydrolysis rate constant for one PDE^*^ subunit and *ν*_*RE*_ is the rate constant for PDE activation by activated rhodopsin.

In a constant background light, when the phototransduction reactions have reached a steady state, the concentrations of phototransduction molecules are invariant:

3 $$\{\begin{array}{c}\frac{d{R}^{\ast }(t)}{dt}=0\Rightarrow {R}^{\ast }=\frac{{{\Phi }}_{BG}}{{k}_{R}}\\ \frac{dPD{E}^{\ast }(t)}{dt}=0\Rightarrow PD{E}^{\ast }=\frac{\,{\nu }_{RE}}{{k}_{E}}{R}^{\ast }=\frac{\,{\nu }_{RE}}{{k}_{E}{k}_{R}}{{\Phi }}_{BG}\\ \frac{dcGMP(t)}{dt}=0\Rightarrow \alpha -({\beta }_{dark}+{\beta }_{sub}\frac{\,{\nu }_{RE}}{{k}_{E}{k}_{R}}{{\Phi }}_{BG})cGMP=0\end{array}$$

The introduction of a competitive PDE inhibitor lowers the average hydrolysis rate constant of cGMP from *β* to *β*_*I*_ according to

4 $${\beta }_{I}=k([I])\beta =\frac{\beta }{1+\frac{[I]}{{K}_{I}}}$$

where *β*_*I*_ is the hydrolysis rate constant of cGMP in the presence of the PDE inhibitor, [*I*] is the concentration of the inhibitor, *k*([*I*]) is a function describing the effect of inhibitor and *K*_*I*_ is the inhibition constant for the inhibitor.

PDE inhibitors decrease the hydrolysis rate of cGMP whereas light increases it. The decrease in the basal PDE activity due to the inhibitor can be compensated for by increasing light. For a steady state in the absence of light and inhibitor,

5 $${\alpha }_{dark}-{\beta }_{dark}cGM{P}_{dark}=0.$$

In the presence of PDE inhibitor and light,

6 $$\alpha -k([I])({\beta }_{dark}+{\beta }_{sub}\frac{\,{\nu }_{RE}}{{k}_{E}{k}_{R}}{{\Phi }}_{BG})cGMP=0.$$

We assume that when the photoreceptor signal is kept constant by increasing the background light intensity during the introduction of the PDE inhibitor, the cGMP concentration in rods does not change. Therefore, the intracellular calcium level and both the cGMP synthesis and hydrolysis rates remain clamped to their dark values. A relation allowing the determination of the basal PDE activity *β*_*dark*_ can be obtained by setting the cGMP concentrations equal in Eqs 5 and 6:

7 $$\begin{array}{rcl}{\alpha }_{dark}-{\beta }_{dark}cGMP & = & {\alpha }_{dark}-k([I])({\beta }_{dark}+{\beta }_{sub}\frac{\,{\nu }_{RE}}{{k}_{E}{k}_{R}}{{\Phi }}_{BG})cGMP\\  & \iff  & {\beta }_{sub}\frac{\,{\nu }_{RE}{K}_{I}}{{k}_{E}{k}_{R}}{{\Phi }}_{BG}={\beta }_{dark}[I].\end{array}$$

The amplification constant *A* is defined as

8 $$A={\nu }_{RE}{\beta }_{sub}{n}_{cGMP},$$

where *n*_*cGMP*_ = 3 is the Hill’s coefficient^2,5^ representing the cooperativity of the cGMP binding sites in the CNG channels^21^. From here we can simplify the equation (7) by applying the amplification constant and by replacing the reaction rate constants of rhodopsin and PDE deactivation with average lifetimes $(\tau =\frac{1}{k})$

9 $${{\Phi }}_{BG}\frac{A{\tau }_{R}{\tau }_{E}}{{n}_{cGMP}}={\beta }_{dark}\frac{[I]}{{K}_{I}}.$$

It is worth noting that the left hand side of the equation (9) corresponds to the PDE activity resulting from the light stimulation in conditions without PDE inhibitor. Thus, the equation can be expressed as

10 $${\beta }_{light}={\beta }_{dark}\frac{[I]}{{K}_{I}}.$$

However, if the inhibitor efficiency towards light-activated and basally activated PDE (*K*_*I*,*dark*_ and *K*_*I*,*light*_) differ, the equation (10) takes the form

11 $${\beta }_{light}={\beta }_{dark}\frac{1+\frac{[I]}{{K}_{I,light}}}{1+\frac{{K}_{I,dark}}{[I]}}.$$

The equation (9) can be used to determine the *β*_*dark*_ from a linear fit to the experimental data where *Φ*_*BG*_ is obtained for every inhibitor concentration [*I*] using cGMP clamp.

#### Modelling flash responses

Phototransduction is well characterized at molecular level and the estimates of rate constants are affirmed by biochemical and electrophysiological analysis^2,3,51^. The state of the art phototransduction models aim at taking into account all the known reactions in phototransduction^52,53^. This, in principle, allows accurate modelling of photoresponses in various conditions but, in practice, leads to a vast number of free parameters. In this study, we utilized a model with as few parameters as possible by disregarding the calcium feedback mechanisms in phototransduction and by simplifying reaction chains to first-order reactions when possible. Similar model is introduced and thoroughly explained in^2^.

We start with the simplifying assumption that after a stimulus impulse, the amount of activated rhodopsin (R^*^) decays according to first-order reaction kinetics

12 $${R}^{\ast }(t,\,C{a}^{2+})={\Phi }{e}^{-\frac{t}{{\tau }_{R}(C{a}^{2+})}},$$

where *Φ* is the number of activated rhodopsins produced by the stimulus flash and *τ*_*R*_ is the average lifetime of activated rhodopsins. The calcium binding protein, recoverin, alters the *τ*_*R*_ in a calcium-dependent manner^54,55^. The activated rhodopsins can activate G-proteins, transducins, they encounter. An activated tranducin (α-subunit) can bind to a PDE molecule and activate it. PDE activation can be assumed to decay with first-order reaction kinetics and thus, the PDE activity can be solved from a convolution

13 $$PD{E}^{\ast }(t,\,C{a}^{2+})={\Phi }{e}^{-\frac{t}{{\tau }_{R}(C{a}^{2+})}}\ast {\nu }_{RE}{e}^{-\frac{t}{{\tau }_{E}}},$$

where *ν*_*RE*_ is the rate constant of PDE^*^ formation by an activated rhodopsin and *τ*_*E*_ is the average lifetime of activated PDE. The rate constant of cGMP hydrolysis is determined by

14 $$\beta (t,\,C{a}^{2+})={\beta }_{dark}+{\beta }_{light}(t,\,C{a}^{2+})={\beta }_{dark}+{\beta }_{sub}PD{E}^{\ast }(t,\,C{a}^{2+}).$$

Furthermore, the rate of cGMP change is determined by the rates of hydrolysis and synthesis of cGMP. The guanylate cyclase activity can be described as a combination of the dark activity, *α*_*dark*_, and the calcium-dependent activity modulation, Δ*α*(*t*, *Ca*^2 +^). Therefore,

15 $$\frac{dcGMP(t)}{dt}={\alpha }_{dark}+{\rm{\Delta }}\alpha (t,\,C{a}^{2+})-({\beta }_{dark}+{\beta }_{light}(t,\,C{a}^{2+}\,))cGMP(t).$$

In GCAPs^−/−^ mice, the Ca^2+^-dependent modulation of cGMP synthesis is removed and Δ*α*(*t*, *Ca*^2+^) = 0. In GCAPs^−/−^ recoverin^−/−^ mice, also the calcium-mediated modulation of rhodopsin lifetime can be disregarded. With the DKO mice, equation (15) simplifies to the form

16 $$\frac{dcGMP(t)}{dt}={\alpha }_{dark}-({\beta }_{dark}+{\beta }_{light}(t))cGMP(t)$$

For a brief moment after the flash stimulus, the rate of cGMP change is dominated by the rate of cGMP hydrolysis by light-activated PDE (−*β*_*light*_(*t*)*cGMP*(*t*)) which surpasses the combined effects from the rate of steady state cGMP synthesis (*α*_*dark*_ = *β*_*dark*_*cGMP*_*dark*_) and basal rate of cGMP hydrolysis (−*β*_*dark*_*cGMP*(*t*)). This can be formulated mathematically as: *β*_*light*_(*t*)*cGMP*(*t*) ≫ *β*_*dark*_(*cGMP*_*dark*_ − *cGMP*(*t*)). Applying the relation to the equation (16) leads to

17 $$\frac{dcGMP(t)}{dt}=-\,{\beta }_{light}(t)cGMP(t).$$

Combined with equations (13 and 14), equation (17) can be used to model the activation phase of the phototransduction. The validity of the equation (17) depends on the phototransduction parameters values and stimulus strength. This will be addressed more in detail in Results-section.

The cation current through CNG channels in rod outer segments obeys Hill’s equation

18 $$\frac{{J}_{cG}}{{J}_{cG,max}}=\frac{cGM{P}^{{n}_{cGMP}}}{cGM{P}^{{n}_{cGMP}}+{{K}_{cGMP}}^{{n}_{cGMP}}}.$$

Here *J*_*cG*_ denotes the current through CNG-channels and *J*_*cG*,*max*_ denotes the maximal current when all the CNG channels are open. The cGMP concentration leading to half maximal channel opening, *K*_*cGMP*_, is in natural conditions always substantially larger than the cGMP level^2^. Thereby the equation (18) simplifies to

19 $$\frac{J(t)}{{J}_{dark}}={(\frac{cGMP(t)}{cGM{P}_{dark}})}^{{n}_{cGMP}},$$

where the *cGMP*_*dark*_ is the concentration of cGMP in dark adapted state and the *J*_*dark*_ is the corresponding value of circulating dark current through CNG channels. In the outer segment region of rods the circulating current *J*(*t*) follows Ohmic relation with the voltage drop in the extracellular space across the rod outer segments, *r*(*t*), which is the signal registered in LERG across the outer segment layer (LERG-OS)^47,56^. The LERG-OS signal normalized by the saturation level, *r*_*max*_, can be considered to follow

20 $$\frac{r(t)}{{r}_{max}}=1-{(\frac{cGMP(t)}{cGM{P}_{dark}})}^{{n}_{cGMP}}.$$

In this model, Eqs 16 and 17 are solved numerically with Matlab. The model assumes that 1) the outer segments are well-stirred, i.e. there are no concentration gradients in different cellular compartments, and 2) protein concentrations do not change significantly during the photoresponse. The model exploiting equation (16) was used for modelling the complete dim flash responses of GCAPs^−/−^ and DKO mice. In addition, we determined the amplification constants and rhodopsin lifetimes for WT, GCAPs^−/−^ and DKO mice by fitting the model exploiting the equation (17) to the beginning of flash response activation phases where calcium mediated modulation can still be regarded insignificant.

## Data Availability

The datasets generated during and/or analysed during the current study are available from the corresponding author on reasonable request.
